# The “garden eel” technique for endoscopic treatment of hepatolithiasis beyond multiple acutely angled sections with strictures

**DOI:** 10.1055/a-2651-0022

**Published:** 2025-08-08

**Authors:** Sho Kitagawa, Narito Murakoshi, Keiya Okamura

**Affiliations:** 1 Department of Hepato-Biliary-Pancreatology, Sapporo Kosei General Hospital, Sapporo, Japan

Advances in balloon-assisted endoscopes and other devices have contributed to the increasing success of endoscopic retrograde cholangiopancreatography (ERCP) in patients with surgically altered anatomy 1 . A recent report suggested that a novel cannula with a movable tip could facilitate bile duct intubation during ERCP in reconstructed intestinal tracts 2 . Here, we report a successful endoscopic treatment using a metal-tipped movable expanded polytetrafluoroethylene (ePTFE) cannula for intrahepatic biliary stones located beyond multiple acutely angled sections with strictures after Roux-en-Y hepaticojejunostomy and left hepatectomy, which could not be approached by other means.

A 50-year-old woman presented with acute cholangitis due to hepatolithiasis. She had undergone extrahepatic bile duct resection with Roux-en-Y hepaticojejunostomy for pancreaticobiliary maljunction and congenital biliary dilatation 33 years previously and left hepatectomy for hepatolithiasis 9 years previously. Percutaneous transhepatic cholangioscopic lithotomy failed because the biliary stones were located beyond an acutely angled branch with strictures ( Fig. 1 ). We then attempted endoscopic treatment using a short-type single-balloon enteroscope (SIF-H290S; Olympus Medical Systems, Tokyo, Japan) and a metal-tipped movable ePTFE cannula (Zeon Medical Inc., Tokyo, Japan) ( Fig. 2 ). By retroflexing the tip of the cannula, we were able to successfully place a guidewire into the acutely angled branch. Moreover, with the help of the tapered metal tip and the flexibility of the cannula, we were able to advance the cannula through the acutely angled branch with strictures that was shaped like an inverted “N.” An additional dilation of the strictures using a fine-gauge balloon catheter was performed, and spontaneous stone passage was observed ( Fig. 3 , Video 1 ).

**Fig. 1:**
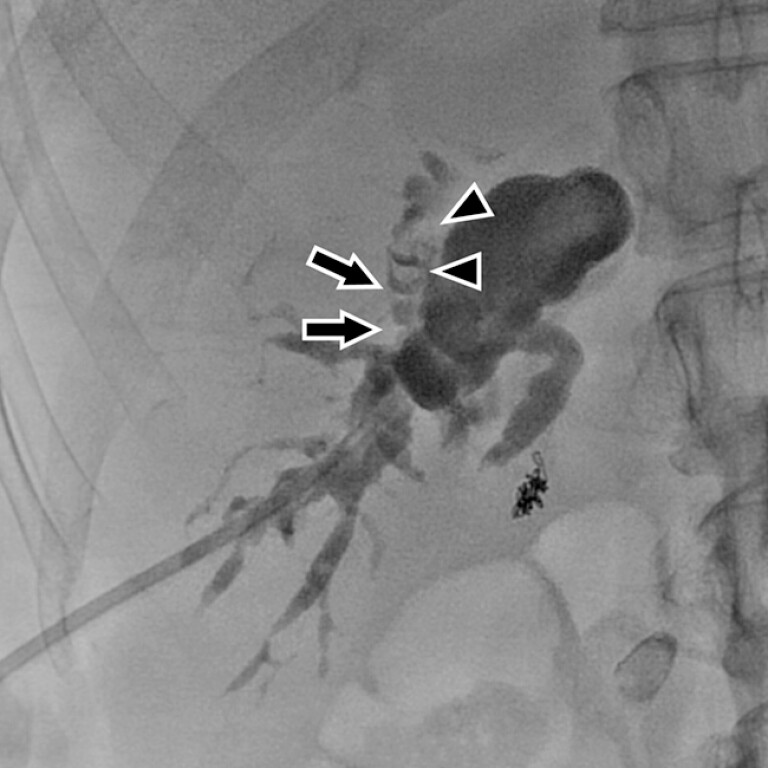
A percutaneous transhepatic cholangiogram (anterior–posterior view) showing hepatolithiasis (arrowheads) in an acutely angled branch with strictures (arrows).

**Fig. 2:**
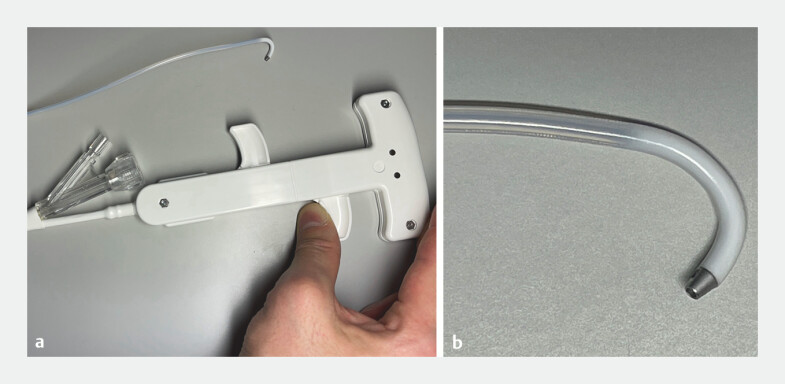
Photograph of the metal-tipped movable expanded polytetrafluoroethylene (ePTFE) cannula (Zeon Medical Inc., Tokyo, Japan) showing: **a** how the tip of the cannula can be moved in the intended direction by grasping its handle; **b** a close up image of the tapered metal tip of the cannula.

**Fig. 3:**
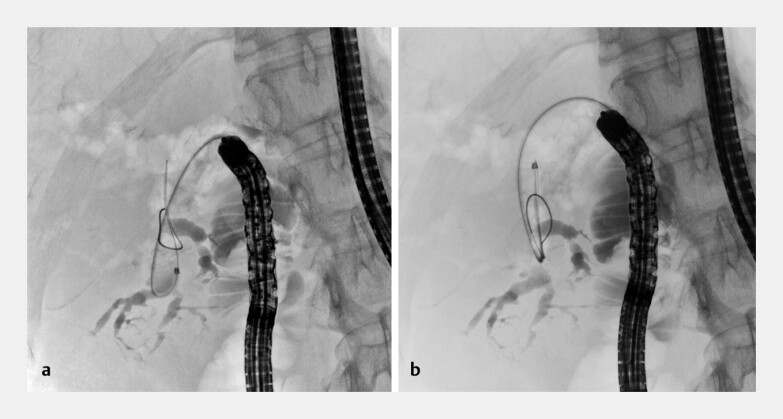
Fluoroscopic images of the endoscopic treatment of complicated hepatolithiasis using the metal-tipped movable cannula showing: **a** a guidewire that had been inserted into the acutely angled branch by retroflexing the tip of the cannula; **b** the cannula after it was advanced through the strictures while still in the retroflexed position.

Endoscopic treatment of hepatolithiasis beyond multiple angled sections with strictures after hepaticojejunostomy using a metal-tipped movable cannula.Video 1

The patient was discharged 2 days after the procedure, without any complications, and no relapse of her cholangitis was observed.

Endoscopy_UCTN_Code_TTT_1AR_2AH

## Notes

Endoscopy E-Videos https://eref.thieme.de/e-videos
*E-Videos* is an open access online section of the journal *Endoscopy* , reporting on interesting cases and new techniques in gastroenterological endoscopy. All papers include a high-quality video and are published with a Creative Commons CC-BY license. Endoscopy E-Videos qualify for HINARI discounts and waivers and eligibility is automatically checked during the submission process. We grant 100% waivers to articles whose corresponding authors are based in Group A countries and 50% waivers to those who are based in Group B countries as classified by Research4Life (see: https://www.research4life.org/access/eligibility/ ). This section has its own submission website at https://mc.manuscriptcentral.com/e-videos .
